# New Appliances and Things Medical

**Published:** 1896-01-04

**Authors:** 


### NEW APPLIANCES AND THINGS MEDICAL.

[We shall be glad to receive, at our Office, 428, Strand, London, W.C., from the manufacturers, specimens of all new preparations and appliances, which may be brought out from time to time.]

### STOUTS AND ALES.

(WHITBREAD AND CO., LIM., 277, GRAY'S INN ROAD, W.C.)

We have lately examined several specimens of stout and ale brewed by the above firm, and have more particularly directed our examination to such points as are of importance from the medical and therapeutic point of view. Although frequently lost sight of, practically all the constituents of these beverages, either singly or combined, are recognised and valued therapeutic agents, and have their place in our pharmacopœia. The list of constituents includes stimulants such as alcohol and inorganic salts, tonics such as the bitter principle, and nutritive bodies such as the malt extractives. In respect to the stouts which we have examined, the malt extractives are present in generous proportion (about 7 per cent.), and the alcoholic contents (about 8 per cent.) entitle it, as far as strength is concerned, to a place among the best examples of its class. The specimens examined include London cooper, half-crown stout, London stout, extra stout, family ale, and pale ale. Though each example may fairly be described as first class of its kind, both as regards purity and quality, the half-crown stout is the one *par excellence* which appeals to us as most likely to be of use to convalescents, invalids, and hard-worked doctors and nurses, since its percentage of malt extractives is a guarantee of high nutritive properties, and its alcoholic contents are sufficient to encourage digestion without dulling physical or mental energy. The price is self-evident from the title.

### NEW HOSPITAL BED-PAN.

It is the ambition of every nurse to improve upon the existing shape and construction of sick-room appliances, and we are glad to welcome any new ideas which make for the greater comfort of the patient and the convenience of the nurse. The improved "Hospital Bed-pan" is the invention of a nurse, Miss Richards, late matron of the Cottage Hospital, Market Drayton, now at the Corbett Hospital, Stourbridge. The principal feature wherein it differs from other articles of the kind is in the lip, whereby emptying of the contents is made easier; it is made, of white porcelain ware, and is well moulded round the inside edge to facilitate cleaning, and the only criticisms we have to make are that the aperture seems somewhat small, and that it would be better if the height were more graduated. The bed-pan can be procured from Messrs. Kirkham, of Stoke, the sole manufacturers. We have no doubt the trifling modifications we have suggested can be easily carried out.

### KUTNOW'S ANTI-ASTHMATIC PREPARATIONS.

(S. Kutnow and Co., 66, Holborn Viaduct, E.C.)

There are very few cases of well-marked spasmodic asthma in which we are not compelled at times to resort to inhalations of sedative drugs in the form of vapour. Though there are many cigarettes and powders for the purpose in the market which have found favour with asthmatics, there is no one form which suits every case. We are, therefore, glad to welcome another variety which will probably extend the usefulness of this form of medication, and to report that Kutnow's anti-asthmatic preparations have proved both pleasant and perfectly harmless, while affording rapid relief in those forms of dyspnœa which are associated with spasm of the bronchioles and certain cases of bronchitis, &c.

### THE RED CROSS NIGHT LIGHT.

We can confidently recommend these night lights, which burn with a clear and steady light. They are made of real wax, consequently last longer in proportion to their size and are more wholesome than the ordinary paraffin substitutes, which are generally used simply because they are slightly cheaper. The cheaper varieties have often a very unpleasant smell in burning, and are, therefore, not so desirable in a sick-room as those which, like the Red Cross night light, are made of wax. Messrs. Francis Tucker, of Kensington, the manufacturers, supply the glasses also, neatly and conveniently arranged, sunk into a cardboard block, this dispensing with saucers or other make-shift arrangements in using the lights.

